# Effect of lipid and cellulose based matrix former on the release of highly soluble drug from extruded/spheronized, sintered and compacted pellets

**DOI:** 10.1186/s12944-018-0783-8

**Published:** 2018-06-09

**Authors:** Madiha Maboos, Rabia Ismail Yousuf, Muhammad Harris Shoaib, Iqbal Nasiri, Tazeen Hussain, Hafiza Fouzia Ahmed, Wajiha Iffat

**Affiliations:** 1 Department of Pharmaceutics, Faculty of Pharmacy and Pharmaceutical Sciences, University of Karachi, Karachi, 75270 Pakistan; 2 Faculty of Pharmacy, Jinnah University for Women, Karachi, 74600 Pakistan

**Keywords:** Atenolol, Pellets, Extended release, Extrusion-Spheronization, Carnauba wax (CW), Glyceryl monostearate (GMS), Hydroxypropyl methylcellulose (HPMC), Ethyl cellulose (EC), Fourier transform spectroscopy (FTIR), Scanning Electron microscopy (SEM)

## Abstract

**Background:**

The study was to develop an extended release (ER) encapsulated and compacted pellets of Atenolol using hydrophobic (wax based and polymeric based) and high viscosity grade hydrophilic matrix formers to control the release of this highly water soluble drug by extrusion/spheronization (ES). *Atenolol* is used for cardiovascular diseases and available as an immediate release (IR) tablet dosage form. The lipids, Carnauba wax (CW), Glyceryl monostearate (GMS) and cellulose based i.e. Hydroxypropyl methylcellulose (HPMC) and Ethyl cellulose (EC) were used in preparing Atenolol ER pellets. Thermal sintering and compaction techniques were also applied to control the burst release of Atenolol.

**Method:**

For this purpose, thirty-six trial formulations (F1-F36) were designed by Response Surface Methodology (RSM), using Design-Expert 10 software, keeping (HPMC K4M, K15 M & K100 M), (EC 7FP, 10FP & 100FP), waxes (GMS, & CW), their combinations, sintering temperature and duration, as input variables. Dissolution studies were performed in pH, 1.2, 4.5 and 6.8 dissolution media. Drug release kinetics using different models such as zero order, first order, Korsmeyer-Peppas, Hixon Crowell, Baker-Lonsdale and Higuchi kinetics were studied with the help of DDsolver, an excel based add-in program.

**Results:**

The formulations F35 and F36 showed compliance with Korsmeyer-Peppas Super case II transport model (R^2^ = 0.975–0.971) in dissolution medium pH 4.5. No drug excipient interaction observed by FTIR. Stereomicroscopy showed that sintered combination pellets, (F35), were highly spherical (AR = 1.061, and sphericity = 0.943). The cross-sectional SEM magnification (at 7000X) of F34 and F35 showed dense cross-linking. The results revealed that the optimized formulations were F35 (sintered pellets) and F36 (compacted pellets) effectively controlling the drug release for 12 h.

**Conclusion:**

Extended-release encapsulated, and compacted pellets were successfully prepared after the combination of lipids CW (10%) and GMS (20%) with EC (10FP 20% & 100FP 20%). Sintering and compaction, in addition, stabilized the system and controlled the initial burst release of the drug. Extended release (ER) Atenolol is an effective alternative of IR tablets in controlling hypertension and treating other cardiovascular diseases.

## Background

Highly soluble drugs are a big challenge for formulation scientists to be designed as an extended release formulations, because of dose dumping, burst release and non-linear release profile [1]. A suitably designed extended release (ER) delivery system can overcome these issues [2]. Through cross-linking, physical interaction of the hydrophilic drug with lipid and cellulose based matrices can be created to form a monolithic system following zero order drug release [3–5].

Glyceryl monostearate (GMS), the monoglyceride of stearic acid, has been reported as an extrusion aid and rate controlling agent, to retard the release of different drugs from various ER dosage forms. Stearates forms an evenly distributed lipid aggregate layer on the surface of matrix former like HPMC, and this interaction with a polymer forms laminated microstructure moisture barrier [6]. Similarly, carnauba wax is another lipid-based ingredient having a high melting point, that also forms a durable super hydrophobic non-polar lipid layer around pellets [7–9]. It is composed of fatty esters (80–85%), free alcohols (10–15%), acids (3–6%), and hydrocarbons (1–3%) [10]. Ethyl cellulose is a thermos-insensitive, hydrophobic inert matrix former [11]. It has been used extensively as a sustained release carrier in pharmaceutical and biomedical industries, due to its good biocompatibility and biodegradability, and in combination with other polymers [12].

Sintering is a densification technique and has been applied to different materials for controlling drug release [13, 14]. Sintering reduces erosion during dissolution, associated with the disintegrating property of microcrystalline cellulose. Materials respond differently at variable temperatures, and percent drug decreases with the rise in sintering temperature [15]. Crack initiation and propagation may occur due to microcrystalline cellulose fibers, which can cause an uncontrolled drug release from weaker and porous pellets. Compaction consolidates the pellets and provides better control on drug release [16].

Atenolol is a cardio-selective beta blocker, widely prescribed as a twice-daily dose for the treatment of hypertension [17] and tachycardia. It is considered as a drug of choice for the prophylaxis of ischemic heart diseases [18, 19]. Atenolol belongs to BCS (Biopharmaceutical Classification System) class III [20] and has poor absorption rate with 50% bioavailability and half-life of 6–8 h only [21, 22].

To date, only Atenolol in hydrophobic and hydrophilic matrix combination, has been evaluated, for extended period release [20, 23–26]. Our preliminary objective was to use Atenolol, as a model highly soluble drug, in waxes (CW and GMS), combined with hydroxypropyl methylcellulose and ethyl cellulose could result in an extended release profile of 12 h. The impact of sintering temperature and duration was also studied on 12-h release profile. However, up to now, no report has been found on this matrix and waxes combination due to the effect of sintering and compaction. Response surface methodology (RSM), Central Composite Design (CCD), was applied to explore the effect of different polymers such as HPMC (K4, 15 and 100 M), EC (7, 10 and 100FP), and waxes (CW and GMS), on the drug release from pellets and their morphology (sphericity and aspect ratio; AR). Thirty-six trial formulations (F1-F36) were designed with the help of Design-Expert version 10 software, keeping polymers, waxes, sintering temperature and duration as input variables, sequential statistical modeling was done to create design space.

## Methods

### Materials

Atenolol was gifted by *Searle Pakistan Limited (SPL)*. Avicel PH101 *(FMC Corporation, USA)*, Glyceryl monostearate (Gattefosse Foundation, Saint-Priest, France), Carnauba wax (BDH Laboratories, England), HPMC (K4, 15 and 100 M), EC (7,10 and 100 FP) (Colorcon, Kent, England), Potassium dihydrogen phosphate, Sodium hydroxide, Methanol (HPLC grade), *Ortho*-phosphoric acid *(Merck, Darmstadt, Germany),* used were of manufacturing and analytical grade and purchased from commercial sources.

### Methods

#### Calculation of extended release dose

A single extended release dose of Atenolol was estimated using 25 mg immediate release dose, The calculated sustained dose of Atenolol for 12 h was found to be 55 mg as per Robinson Eriksson equation and the reported pharmacokinetic data [27, 28]. Pharmacokinetic studies show that 25 mg of Atenolol produce expected therapeutic effects within 2 h with a half-life of 6 h. Thus, the first order overall elimination rate constant was calculated to be,

1 $$ \boldsymbol{ke}=\mathbf{0.693}/\mathbf{6}=\mathbf{0.1155}\ \boldsymbol{mg}/\boldsymbol{h} $$

The availability rate was,

2 $$ \boldsymbol{R}=\boldsymbol{KeD}=\mathbf{0.1155}\times \mathbf{25}=\mathbf{2.8875}\ \boldsymbol{mg}/\boldsymbol{h} $$

Where *D* is the initial dose of the drug. The maintenance dose *D*_m_ was calculated as

3 $$ {\boldsymbol{D}}_{\boldsymbol{m}}=\boldsymbol{Rh}=\mathbf{2.8875}\times \mathbf{12}=\mathbf{34.65}\ \boldsymbol{mg} $$

Where h is the number of hours for which sustained action is desired.

Thus, $$ \boldsymbol{Total}\ \boldsymbol{Dose}=\boldsymbol{D}+\boldsymbol{Dm}=\mathbf{25}+\mathbf{34.65}=\mathbf{59.56} $$

4 $$ {\boldsymbol{D}}_{\boldsymbol{corrected}}=\boldsymbol{D}-{\boldsymbol{Rt}}_{\boldsymbol{p}}=\mathbf{59.65}-\mathbf{2.8875}\times \mathbf{2}=\mathbf{53.875}\boldsymbol{mg}\ \left(\approx \mathbf{55}\boldsymbol{mg}\right) $$

Where, t_P_ is the time required to achieve a peak plasma level. Hence, an oral controlled release formulation of Atenolol should contain a total dose of 53.875 mg (≈55 mg).

#### Selection of granulating fluid

To prepare spherical pellets, water alone and water-ethanol mixture (1:1), were tested as granulating fluids separately, however, pellets with good sphericity and maximum yield were obtained with water [29].

#### Response surface methodology (RSM)

The most commonly used RSM technique, Central Composite Design (CCD) was applied by Design-Expert 10 (State-Ease, Inc., USA.). The independent variables were hydroxypropyl methylcellulose (HPMC K4M, K15 M & K100 M), ethyl cellulose (EC 7FP, 10FP &100FP), waxes (GMS, & CW), sintering temperatures (70^°^C, 80^°^C and 90^°^C) and duration (60, 90 and 120 s). Their effects were observed on the percentage drug release (1st hour) and morphology (Aspect Ratio; AR) of pellets, as critical parameters. Factor-response relationship was elaborated by the help of a mathematical model and the significance of model was estimated by analysis of variance (ANOVA). The closer the value of the coefficient of determination (*r*^*2*^) to unity, better would be the model fitting. The model was selected on the basis of minimum values (closer to zero) of standard deviation (SD) and predicted residual error sum of squares (PRESS) that further cross validated the appropriateness of suggested model. The perturbing effect of input variable on critical parameters was shown by perturbation plot and 3D response surface plot was used to exhibit the relationship between the variables.

#### Preparation of pellets

According to the composition of trial batches (Table 1), formulation ingredients were weighed accurately and mixed together for 15 min in a poly bag. Wet mass of suitable consistency was obtained in a low shear planetary mixer (Kenwood Chef, Hampshire, UK) by adding water as a granulating fluid. The mass was then subjected to 1 mm screen of the extruder (mini screw laboratory scale, Caleva, Process solution Ltd., Model. M.S.E, Dorset, UK). The extrudates were then immediately transferred to a multi-bowl bench top spheronizer (Caleva, Process solution Ltd., Model M.B.S 120, Dorset, UK) at 800–1000 rpm for 15 min. The speed (rpm) and time of spheronization were adjusted for obtaining the spherical granules. The pellets were dried overnight (8 h) at 35°C and then screened through 18 mesh sieve. The recovery of dried pellets was calculated by the following equation [30].

**Table 1:** The Composition of Atenolol ER matrix pellets formulation

FORMULATIONS		mg (%)										
	Drug	HPMC K4M	HPMC K15M	HPMC K100M	GMS	CW	EC 7FP	EC 10FP	EC 100FP	MCC	SINTERING	Total
F00	55(45.8)									65(54.1)		120(100)
F0	55(45.8)									65(54.1)		120(100)
F1	55(45.8)	6(5)								59(49.2)		120(100)
F2	55(45.8)	24(20)								41/34.2		120(100)
F3	55(45.8)		24(20)							41(34.2)		120(100)
F4	55(45.8)			24(20)						41(34.2)		120(100)
F5	55(45.8)				12(10)					54(45)		120(100)
F6	55(45.8)				24(20)					41(34.2)		120(100)
F7	55(45.8)				36(30)					29(24.2)		120(100)
F8	55(45.8)				48(40)					17(14.2)		120(100)
F9	55(45.8)				60(50)					5(4.2)		120(100)
F10	55(45.8)					6(5)				54(45)		120(100)
F11	55(45.8)					12(10)				54(45)		120(100)
F12	55(45.8)					12(10)				54(45)		120(100)
F13	55(45.8)					18(15)				47(39.2)		120(100)
F14	55(45.8)					24(20)				41(34.2)		120(100)
F15	55(45.8)					24(20)				41(34.2)		120(100)
F16	55(45.8)	24/20			12(10)					29(24.2)		120(100)
F17	55(45.8)				36(30)	12(10)				17(14.2)		120(100)
F18	55(45.8)				36(30)	12(10)				17(14.2)		120(100)
F19	55(45.8)						24(20)			41(34.2)		120(100)
F20	55(45.8)							24(20)		41(34.2)		120(100)
F21	55(45.8)								24(20)	41(34.2)		120(100)
F22	55(45.8)				24(20)	12/10	12(10)			17(14.2)		120(100)
F23	55(45.8)				24(20)	12(10)	18(15)			11(9.2)		120(100)
F24	55(45.8)				24(20)	12(10)	24(20)			5(4.2)		120(100)
F25	55(45.8)				24(20)	12(10)		6(5)		23(19.2)		120(100)
F26	55(45.8)				24(20)	12(10)		12(10)		17(14.2)		120(100)
F27	55(45.8)				24(20)	12(10)		18(15)		11(9.2)		120(100)
F28	55(45.8)				24(20)	12(10)		24(20)		6(5)		120(100)
F29	55(45.8)				24(20)	12(10)			6(5)	23(19.2)		120(100)
F30	55(45.8)				24(20)	12(10)			12(10)	17(14.2)		120(100)
F31	55(45.8)				24(20)	12(10)			12(10)	11(9.2)	√	120(100)
F32	55(45.8)				24(20)	12(10)			18(15)	6(5)		120(100)
F33	55(45.8)				24(20)	12(10)			18(15)	6(5)	√	120(100)
F34	55(45.8)				24(20)	12(10)			24/20			120(100)
F35	55(45.8)				24(20)	12(10)			24/20		√	120(100)

5 $$ \boldsymbol{Yield}\%=\frac{\boldsymbol{Actual}\ \boldsymbol{weight}\ \boldsymbol{of}\boldsymbol{pellets}}{\boldsymbol{Theoratical}\ \boldsymbol{weight}\ \boldsymbol{of}\ \boldsymbol{pellets}}\boldsymbol{X}\ \mathbf{100} $$

#### Sintering and compaction of pellets

Based on the drug release profile selected batches were subjected to sintering and compaction. Thermal treatment was done at 70^°^C, 80^°^C and 90^°^C for the duration of 60, 90 and 120 s in a hot air oven. Best sintered pellets were obtained at 90^°^C for 120 s [15, 31–33].

Formulation F28 (GMS 20%, CW 10% and EC 10FP 20%) was selected for compaction. The accurately weighed quantity of pellets containing Atenolol (55 mg) was compressed with Microcrystalline cellulose (as a diluent) by direct compression method on a single punch tablet machine (Korsch, Erweka, Frankfurt Germany), using flat shape punches [34]. The compression forces were kept variable, ranging from 3-7Kg. Finally, the compression force selected was 5 Kg, to compact the pellets in the form of a tablet (F36), with suitable strength (hardness) and an average weight of (130 mg).

#### Fourier transform infrared spectroscopy (FTIR):

Compatibility and interaction of the pure drug with waxes and polymers was determined by FTIR (Thermo Nicolet Avatar, 330). Infrared spectra of pure drug and formulations were recorded over wave numbers ranging from 4500 to 1000 cm^− 1^ [35].

#### Flow characterization and compressibility index:

Samples of 20 g of pellets were put into 200 mL graduated cylinder. Bulk density, tap density, Carr’s index and Hausner’s ratio, were calculated [36, 37].

##### Angle of repose

The angle of repose of all formulations of Atenolol and Glyburide was determined by the fixed base method, using following formula;

6 $$ \boldsymbol{tan}\boldsymbol{\theta } =\raisebox{1ex}{$\boldsymbol{height}$}\!\left/ \!\raisebox{-1ex}{$\mathbf{0.5}\boldsymbol{base}$}\right. $$

##### Bulk density

Bulk Densities of pellets were determined, by pouring 10 g of pellets in a graduated cylinder, then bulk volumes were measured in ml and bulk densities were calculated.

7 $$ \boldsymbol{Bulk}\ \boldsymbol{Density}=\frac{\boldsymbol{Mass}}{\boldsymbol{Bulk}\ \boldsymbol{Volume}} $$

##### Tapped density

Tapped density was determined by tapping the graduated cylinder (till no further reduction in volume was observed). Tapped density was then calculated as follows

8 $$ \boldsymbol{Tapped}\ \boldsymbol{Density}=\frac{\boldsymbol{Mass}}{\boldsymbol{Tapped}\ \boldsymbol{Volume}} $$

##### Carr’s index

Compressibility index can be used to predict the flow property and is based on the density measurements. Carr’s index was calculated using the following equation.

9 $$ \boldsymbol{Car}{\boldsymbol{r}}^{\prime}\boldsymbol{s}\ \boldsymbol{index}\ \left(\%\right)=\frac{\left(\boldsymbol{Tapped}\ \boldsymbol{Density}-\boldsymbol{Poured}\ \boldsymbol{Density}\right)}{\boldsymbol{tapped}\ \boldsymbol{Density}}\boldsymbol{X}\ \mathbf{100} $$

##### Hausner’s ratio

Flowability of pellets was determined by Hausner using the following equation.

10 $$ \boldsymbol{Hausne}{\boldsymbol{r}}^{\prime}\boldsymbol{s}\ \boldsymbol{Ratio}=\frac{\boldsymbol{Tapped}\ \boldsymbol{Density}}{\boldsymbol{Poured}\ \boldsymbol{Density}} $$

#### Quality evaluation of compacted pellets

Pharmaceutical quality such as uniformity of weight, hardness, friability, and disintegration of compacted pellets (F36) were evaluated as per pharmacopeial specification [38].

The weight of 20 compacted pellets (Tablets) was observed using analytical weighing balance (Sartorius, Goettingen, Germany), Tablet Hardness (crushing strength) using hardness tester (OSK-Fujiwara Seiki, Japan) and Friability Test was performed on Roche type friabilator (Erweka, Husenstamm, Germany). Disintegration time was observed for 6 tablets using USP Disintegration apparatus (Erweka, Heusenstamm, Germany).

#### Encapsulation of pellets

The weight of pellet formulations (F00-F35), holding the accurate amount of the sustained dose of Atenolol (55 mg), was 120 mg, that was encapsulated directly into the hard gelatin capsule size of 00.

#### Assay of compacted and encapsulated pellets

##### Sample preparation

An average weight of one capsule was taken after weighing 10 capsules from each formulation. Pellets were triturated using Mortar and pestle making the final strength of 30 μg/ml of Atenolol in Methanol. Glyburide was taken as an Internal standard with a concentration of 15 μg/ml of Glyburide, content was sonicated with Digital Ultrasonic Cleaner (Supersonic X3, Germany) for 15 min, then the volume was made up in 25 ml volumetric flask. The same procedure was carried out to prepare reference standard solutions of Atenolol and Glyburide. The same procedure was adopted for compacted pellets where 10 tablets were crushed with the help of Mortar and pestle.

##### Mobile phase

The mobile phase was prepared by the addition of 20 parts of distilled water in 80 parts of HPLC grade Methanol (Merck KGaA, Darmstadt, Germany) and pH was adjusted to 3.4 ± 0.2 with Ortho-Phosphoric acid (Merck KGaA, Darmstadt, Germany). The mobile phase was filtered using 0.45 μ filter and sonicated with Digital Ultrasonic Cleaner (Supersonic X3, Germany).

##### Chromatographic condition

An isocratic high performance liquid chromatographic method using Shimadzu HPLC system having pump LC-10AT VP and SPD-10AVP as UV detector (Shimadzu Corp. Kyoto, Japan) with C18 column (5um, 250 × 4.6 mm) (Phenomenex, Torrance, USA) was used for separating and estimating drug content. The flow rate was 1 ml/min and the volume of injection was 20 μl. The wavelength of detection was 235 nm.

Limits: 90–110%.

#### In vitro drug release study

The dissolution profiles of formulations (F00-F35) were generated by performing multiple point dissolution studies in 900 ml of pH 1.2, 4.5 and 6.8 buffers as a dissolution media using USP dissolution apparatus I (Erweka, Heusenstamm, Germany) at 50 rpm. Ten ml of sample were drawn at 0.5, 1, 2,3,4,5, 6, 7, 8, 9, 10, 11 and 12 h and sink condition was maintained. The percentage drug release was determined on UV-VIS Spectrophotometer (UV- 1800 Shimadzu Corp, Japan) at λ_max_ = 275 nm. USP dissolution apparatus II was used to study the dissolution profile of formulation F36 (compressed pellets) only, under the same dissolution conditions [39, 40].

#### Release kinetics and mechanism:

Drug release kinetics and mechanism of successful formulations were assessed using DDSolver, an Excel based Add-in program for the following models [41]. In these models, ‘F’ is the amount of drug released at time ‘t’, and ‘k’ is a dissolution rate constant.

- *Model I: First order kinetics*

11 $$ \boldsymbol{F}=\mathbf{100}\left(\mathbf{1}-{\boldsymbol{e}}^{-\boldsymbol{kt}}\ \right) $$

Drug release is proportional to the amount of drug remaining in dosage form so that the amount of drug release diminishes per unit of time.

- *Model II: Zero-order kinetics*

Drug dissolution from rate-controlled dosage forms are best explained by zero order kinetics. It can be expressed as,

12 $$ {\boldsymbol{Q}}_{\boldsymbol{t}}={\boldsymbol{Q}}_{\boldsymbol{o}}+{\boldsymbol{K}}_{\boldsymbol{o}}\boldsymbol{t} $$

Where Q_t_ is the amount of drug dissolved in time t, Q_0_ is the initial amount of drug in the solution and K_0_ is the zero order release constant expressed in units of concentration /time [42].

- *Model III: Higuchi Kinetics*

13 $$ \kern2.25em \boldsymbol{F}=\boldsymbol{k}\sqrt{\boldsymbol{t}} $$

To determine the process of diffusion through a reasonably intact matrix Higuchi model was applied [43].

- *Model IV: Baker and Lonsdale Model*

14 $$ \boldsymbol{kt}=\mathbf{2}/\mathbf{3}\left[\mathbf{1}-{\left(\mathbf{1}-\boldsymbol{F}\right)}^{\raisebox{1ex}{$\mathbf{2}$}\!\left/ \!\raisebox{-1ex}{$\mathbf{3}\kern0.75em $}\right.}\right]-\boldsymbol{F} $$

The model was applied to know the release control of drug from the matrix, if the matrix was homogeneous and/or not fractured or containing no capillaries, could affect the amount and rate of drug release [44].

- *Model V: Hixson and Crowell Model*

15 $$ \boldsymbol{F}=\mathbf{100}\left[\mathbf{1}-{\left(\mathbf{1}-\boldsymbol{kt}\right)}^{\mathbf{3}}\right] $$

This equation is applicable to evaluate the dissolution behavior of uniformly sized particles like pellets and was used to study the effect of surface area on the dissolution of drug from matrix [45].

- *Model VI: Korsmeyer and Peppas Model*

The mode was used to study the drug release mechanism and rate of drug release as a function of time.

16 $$ \boldsymbol{F}=\boldsymbol{k}{\boldsymbol{t}}^{\boldsymbol{n}} $$

Where,

**Table:** 

Release exponent (*n*)	Drug Release mechanism	Rate as a function of time
0.5	Fickian diffusion	*t* ^-0.5^
0.5 < *n* < 1.0	Anomalous Transport	*t* ^n-1^
1.0	Case-II transport	Zero order release
Higher then 1.0	Super Case-II Transport	*i* ^n-1^
Interpretation of diffusional release mechanisms from polymer [46–48]		

#### Image analysis of pellets:

Photomicrographs of pellets (*n* ≥ 50) were taken with a stereomicroscope (Amscope Digital, LED-1444A, USA) at 10X magnification, to obtain top light illumination of the pellets against a dark surface. Images were analyzed by image analysis software (NIH Image J 1.47v, USA). Samples of each batch was characterized by means of Feret diameter (*e*_*R*_) (Eq.17) (average of 180 caliper measurements with an angle of rotation of 1°), aspect ratio (AR) (Eq.10) (ratio of longest Feret diameter and its longest perpendicular diameter) and two-dimensional shape factor or sphericity (Eq.19) [49].

17 $$ {\boldsymbol{e}}_{\boldsymbol{R}}=\frac{\mathbf{2}\boldsymbol{\pi }}{\boldsymbol{P}}\frac{{\boldsymbol{r}}_{\boldsymbol{e}}}{\boldsymbol{f}}-\sqrt{\mathbf{1}-{\left(\frac{\boldsymbol{b}}{\mathbf{1}}\right)}^{\mathbf{2}}} $$

Where r is the radius, P_m_ is the perimeter, l is the length (longest Feret diameter) and b is the breadth (longest diameter perpendicular to the longest Feret diameter) of a pellet. An average value for all pellets was calculated as the mean pellet size (mean FD).

18 $$ \boldsymbol{Sphericity}=\frac{\mathbf{4}\boldsymbol{\pi } \boldsymbol{A}}{{\mathbf{\mathcal{P}}}_{\boldsymbol{m}}^{\mathbf{2}}} $$

19 $$ \boldsymbol{Aspect}\ \boldsymbol{ratio}=\frac{\boldsymbol{dmax}}{\boldsymbol{dmin}} $$

Where *d*_max_ and *d*_min_ were the longest and shortest Feret diameters measured respectively.

#### Surface morphology:

Morphology of the pellets was determined by SEM (JSM-6380A, Jeol, Japan). Pellets were coated with a gold film by Auto Coater (JFC-1500, Jeol, Japan) to assure conductivity, and scanning was performed under different magnifications ranging from 93,500 to 911,000 at 16 kV voltage. Photomicrographs were taken with a scanning electron microscope and measured at four sites per pellet.

#### Stability studies:

Extended release optimized formulation (F35 and F36) were stored for 12 months at 40 ± 2 °C/75% ± 5% RH (relative humidity), as per the guidelines of International Conference on Harmonization (ICH). Sintered encapsulated (F35) and compacted pellets (F36) were placed in amber glass bottles and stored in a humidity chamber (Nuaire, USA). Samples were drawn every 3 months and their physical appearance, drug content and release characteristics in different dissolution medium i.e. pH 1.2, 4.5 and 6.8 were determined. The shelf life of optimized formulation was calculated using shelf life calculation feature of Minitab software version 17 [50].

## Results

### Selection of granulating fluid

Initially, two formulations (F00 and F0) were prepared (Table 1) with the different composition of granulating fluid i.e. water alone and a mixture of water and ethanol (1:1) respectively. Sphericity and better yield were obtained by using water alone as a granulating fluid.

### Response surface methodology

**Fig. 1:**
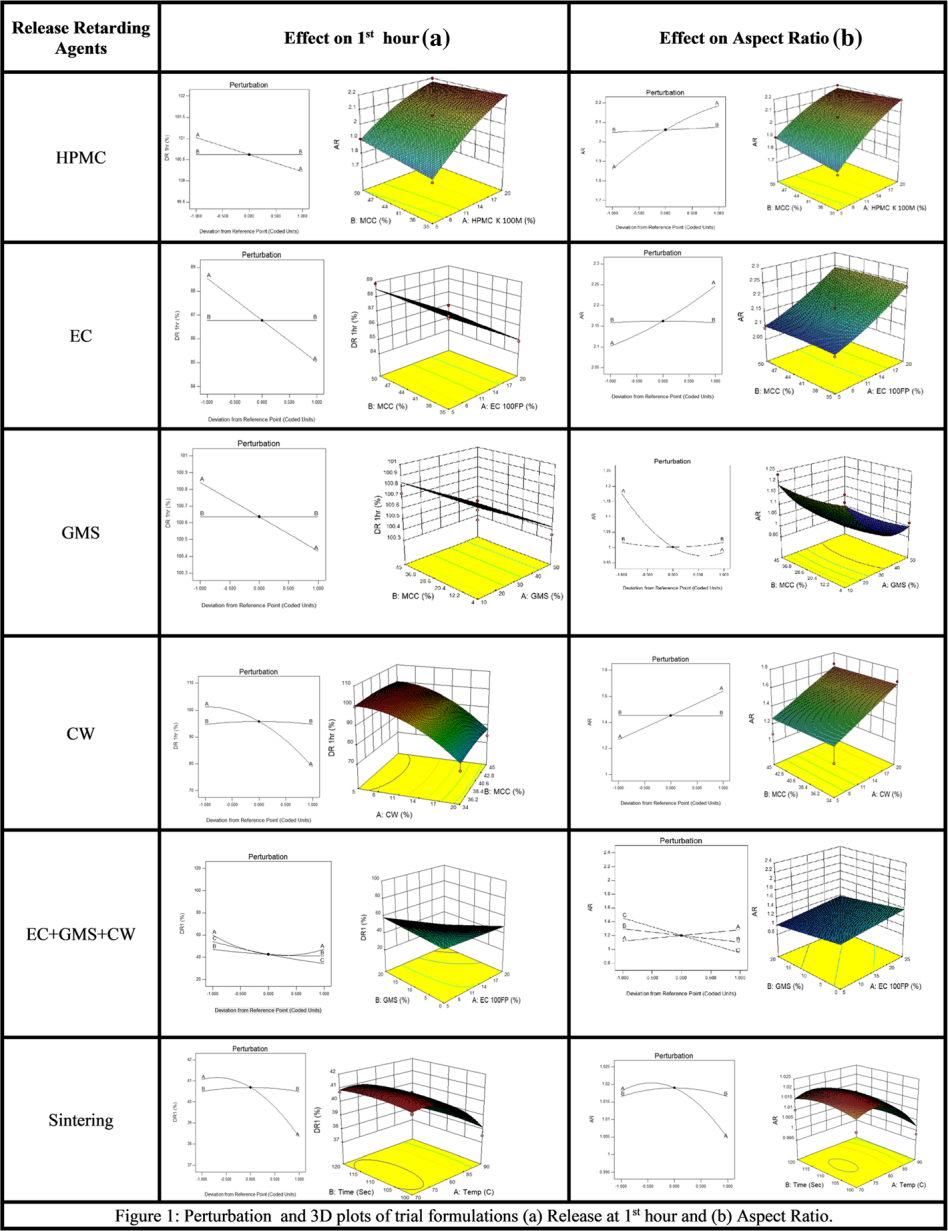
Perturbation and 3D plots of trial formulations (**a**) Release at 1st hour and (**b**) Aspect Ratio

Figure 1 (a), shows the perturbing effect of input variables on drug release at 1st hour and aspect ratio (AR). Lipids (CW and GMS) and polymers (HPMC and EC), when used alone and in combination, influenced the atenolol release. The presence of microcrystalline cellulose (MCC), however, did not affect the rate of drug release. The rate of dissolution was observed to be affected by the rise in sintering temperature, contrarily, the duration of thermal treatment did not change the drug release evidently. The 3D plots of Fig. 1 (a), shows that when used alone or in combination, the maximum concentrations of high viscosity grades of polymers (HPMC K100 M& EC100 FP) and waxes (GMS & CW), could not control the release of atenolol, and maximum drug release was found within 1st hour.

**Fig. 2:**
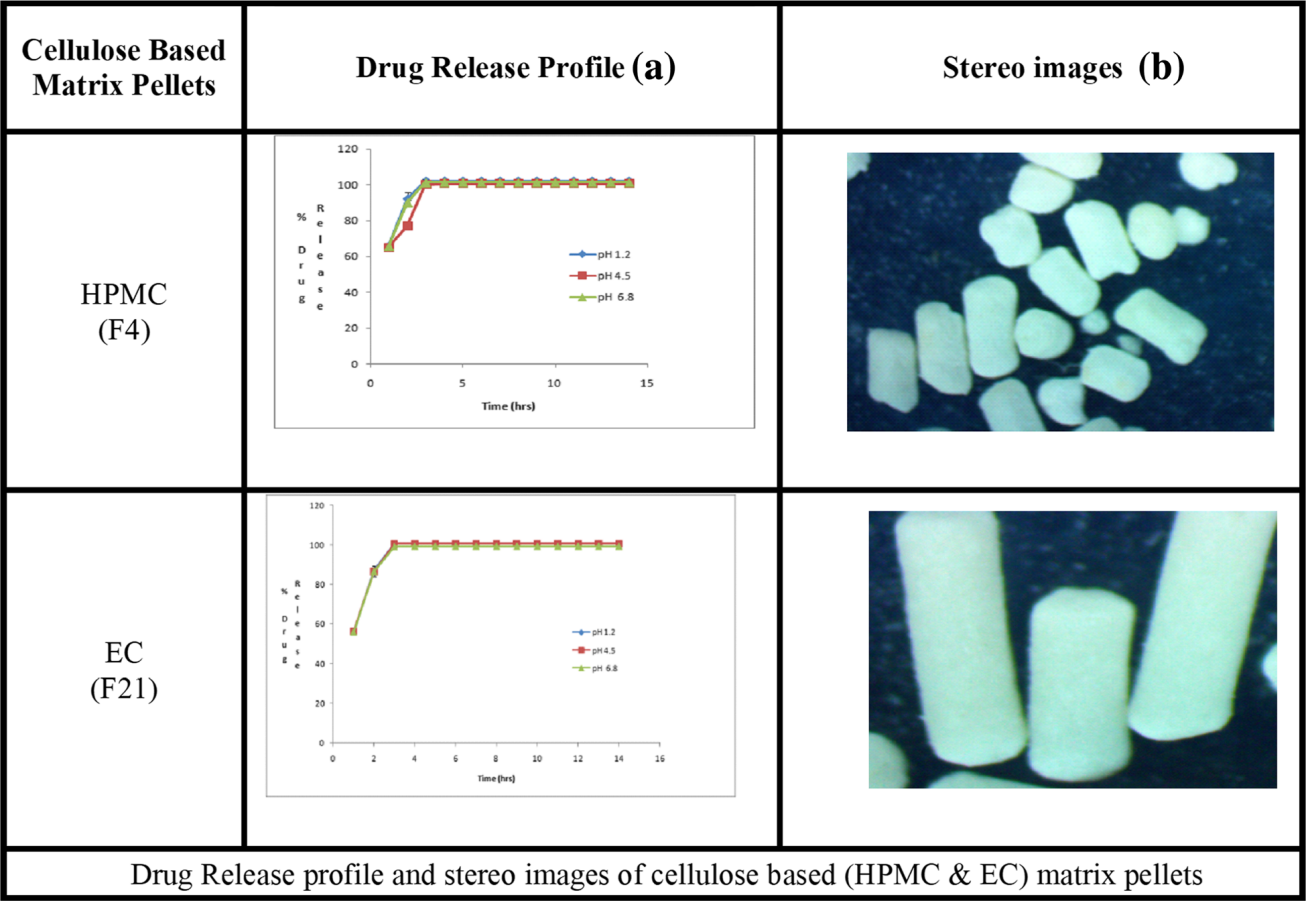
Drug Release profile and stereomicroscopic images of cellulose based (HPMC & EC) matrix pellets

**Fig. 3:**
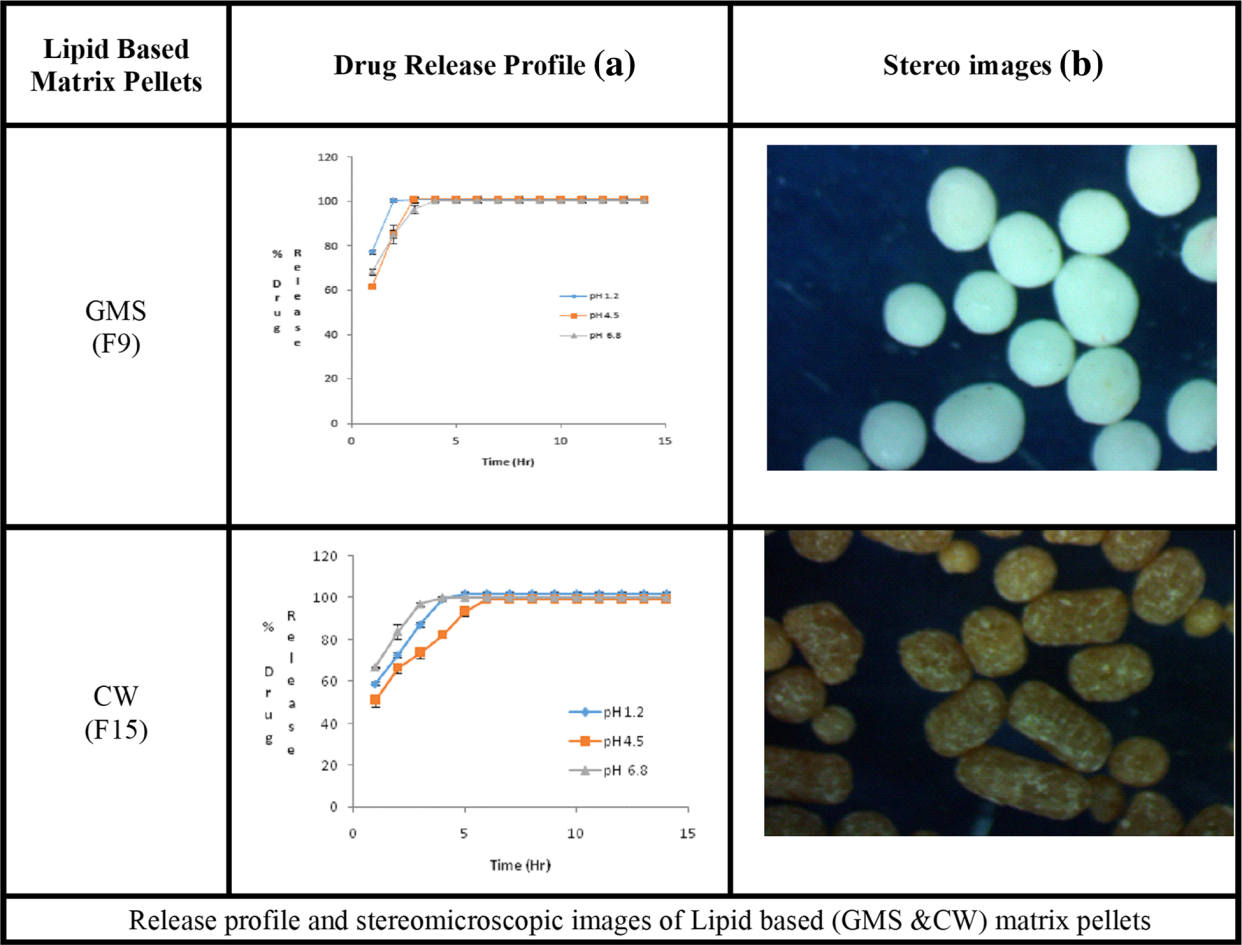
Release profile and stereomicroscopic images of Lipid based (GMS &CW) matrix pellets

**Fig. 4:**
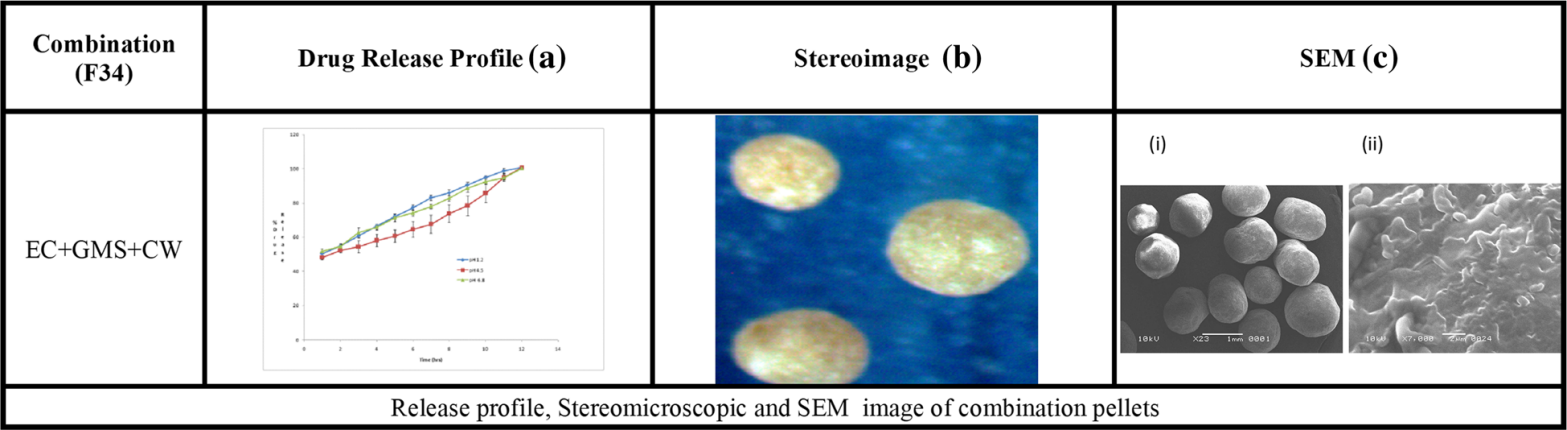
Release profile, Stereomicroscopic and SEM image of combination pellets

**Fig. 5:**
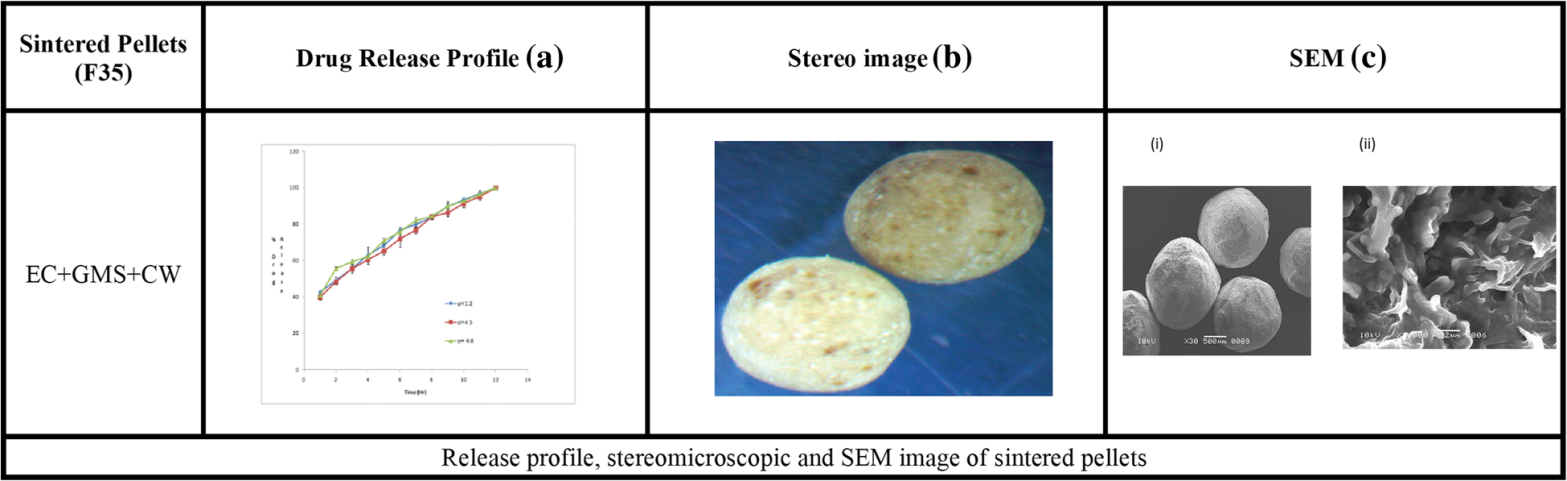
Release profile, stereomicroscopic and SEM image of sintered pellets

Figure 1 (b) depicts that Aspect ratio (AR) also changed with the concentrations of polymers and waxes. The pellets prepared by using water as a granulating fluid had better sphericity and AR (0.951; 1.052) than pellets prepared by ethanol and water mixture (0.938, 1.066). Pellets containing HPMC and EC had poor morphology i.e. (AR = 1.764–2.517, sphericity = 0.396–0.608) and (AR = 1.412–1.756, sphericity = 0.647–0.752) respectively. The best shape was obtained at the maximum concentration of GMS (AR = 1.017, sphericity = 0.984), but pellets containing CW had poor AR (1.268–2.483) and sphericity (0.403–0.655) (Table 2,). The stereo images showing the morphology of atenolol pellets are presented in Figs. 2b and 3b.The stereo-micrographs (Fig. 4b) of the combination pellet (F34), exhibited good surface morphology (AR = 1.051, and sphericity = 0.951). Sintering did not affect the shape of the pellets (AR = 1.061, and sphericity = 0.943) (Fig. 5b). However, the effect of concentration of MCC on the aspect ratio (AR), remained negligible, but sintering temperature, unlike duration of thermal treatment, showed the perturbing effect on it. Atenolol pellets were subjected to physical testing like bulk density, tapped density, angle of repose, Carr’s index, and Hausner’s ratio, and results were obtained within the official range as per USP NF30. Percent assay of pellet formulations (F00-F35) were determined and found in compliance with the official reference range (90-110%) (Table 3).

**Table 2:** Image analysis of matrix and sintered formulations

Formulations	Area	Perimeter	Circumference	Feret Diameter	Aspect Ratio	Sphericity
F00	37,632	688.009	0.999	226.883	1.066	0.938
F0	30,484	618.894	1	202.988	1.052	0.951
F1	15,453	545.717	0.652	187.182	1.764	0.567
F2	18,006	561.265	0.718	202.41	1.928	0.519
F3	23,448	689.207	0.62	254.167	2.517	0.397
F4	13,765	502.334	0.685	177.505	1.646	0.608
F5	13,502	413.119	0.994	142.79	1.174	0.852
F6	8957	336.15	0.996	113.745	1.119	0.894
F7	12,079	389.557	1	127.883	1.048	0.954
F8	9258	340.863	1	111.018	1.027	0.973
F9	11,516	380.133	1	123.045	1.017	0.984
F10	13,208	444.987	0.838	158.028	1.528	0.655
F11	11,243	413.361	0.827	140.46	1.268	0.789
F12	16,118	516.55	0.759	179.477	1.689	0.592
F13	17,678	575.009	0.672	229.369	2.364	0.423
F14	18,515	592.862	0.662	231.683	2.483	0.403
F15	26,743	656.143	0.781	258.606	1.883	0.531
F16	25,230	626.354	0.808	234.196	1.694	0.59
F17	11,738	384.845	0.996	131.746	1.149	0.87
F18	8371	326.726	0.985	117.516	1.286	0.778
F19	12,975	457.073	0.78	154.311	1.412	0.708
F20	9916	412.01	0.734	123.794	1.605	0.752
F21	6586	287.456	1	94.895	1.756	0.647
F22	6643	289.027	0.999	95.885	1.067	0.937
F23	6988	301.593	0.965	114.438	1.458	0.686
F24	7010	296.881	0.999	98.858	1.078	0.928
F25	7952	317.301	0.993	110.653	1.196	0.836
F26	11,208	375.42	0.999	123.794	1.059	0.944
F27	8952	337.721	0.986	120.702	1.263	0.792
F28	8906	334.58	1	108.005	1.008	0.992
F29	9001	336.15	1	108.268	1	1
F30	13,271	408.407	1	131.947	1.014	0.986
F31	11,703	383.274	1	124.04	1.017	0.983
F32	9238	340.863	0.999	114.739	1.106	0.904
F33	13,901	417.832	1	135.831	1.029	0.971
F34	11,872	386.416	0.999	127.012	1.051	0.951
F35	8175	320.442	1	105.802	1.061	0.943

**Table 3:** Physical and chemical evaluation of matrix and sintered pellet formulations

Formulations	Bulk Density g/ml	Tapped Density g/ml	Angle of Repose *θ*	Carr’s Index %	Hausner’s Ratio	Assay %
F00	0.625	0.704	23.08	11.222	1.126	101.481
F0	0.557	0.597	21.23	6.700	1.072	101.373
F1	0.675	0.709	52.19	4.795	1.050	101.872
F2	0.687	0.712	53.23	3.511	1.036	100.998
F3	0.654	0.698	54.18	6.304	1.067	101.623
F4	0.769	0.806	55.54	4.591	1.048	100.749
F5	0.826	0.931	27.56	11.278	1.127	99.987
F6	0.8	0.967	25.27	17.270	1.209	98.765
F7	0.654	0.765	26.15	14.510	1.170	101.675
F8	0.554	0.578	26.56	4.152	1.043	100.765
F9	0.567	0.6	29.54	5.500	1.058	100.653
F10	0.674	0.713	27.56	5.470	1.058	99.765
F11	0.598	0.67	27.89	10.746	1.120	99.876
F12	0.479	0.501	28.98	4.391	1.046	97.676
F13	0.675	0.698	30.23	3.295	1.034	100.109
F14	0.775	0.891	30.78	13.019	1.150	100.657
F15	0.765	0.865	31.78	11.561	1.131	101.762
F16	0.567	0.613	29.76	7.504	1.081	101.765
F17	0.767	0.897	26.67	14.493	1.169	100.765
F18	0.765	0.887	28.56	13.754	1.159	100.675
F19	0.897	0.953	32.33	5.876	1.062	101.876
F20	0.785	0.866	32.87	9.353	1.103	101.767
F21	0.764	0.824	33.32	7.282	1.079	98.765
F22	0.745	0.867	28.56	14.072	1.164	99.762
F23	0.699	0.765	28.76	8.627	1.094	100.765
F24	0.776	0.845	26.87	8.166	1.089	99.765
F25	0.798	0.878	30.76	9.112	1.100	98.766
F26	0.687	0.786	27.67	12.595	1.144	99.765
F27	0.657	0.759	27.56	13.439	1.155	101.673
F28	0.765	0.823	28.65	7.047	1.076	100.783
F29	0.734	0.845	25.22	13.136	1.151	100.786
F30	0.765	0.876	25.87	12.671	1.145	101.783
F31	0.734	0.823	23.78	10.814	1.121	100.672
F32	0.71	0.83	25.78	14.458	1.169	100.37
F33	0.76	0.9	27.4	15.556	1.184	100
F34	0.83	0.95	28.7	12.632	1.145	99.87
F35	0.9	1.11	27.8	18.919	1.233	100.12

### Effect of polymers and waxes on drug release

In all dissolution media (pH 1.2, 4.5 and 6.8), formulations containing highest concentrations of HPMC K100 M (F4) and EC 100FP (F21), remained unable to control the atenolol release, for an extended period (12 h) and maximum drug released was obtained within 5 h (Fig. 2a). Even the lipid-based matrix formers in their highest concentrations (GMS 50% and CW 20%) failed to control the atenolol release from pellets (Fig. 3a). The combination pellets (F34), exhibited drug release control up to 12 h but initial burst release was observed in 1st hour (50.3% at pH 1.2, 48.14% at pH 4.5 and 51.79% at 6.8) as shown in Fig. 4a.

### Effect of sintering

The burst release at first hour was controlled after the thermal treatment at the optimized temperature and duration of 90 °C and 120 s. after thermal treatment, the release was found to be reduced from 50.30 to 42.40%, 48.14 to 39.67% and 51.79 to 40.67% at pH 1.2, 4.5 and 6.8 respectively (Fig. 5a).

### Effect of compaction

**Fig. 6:**
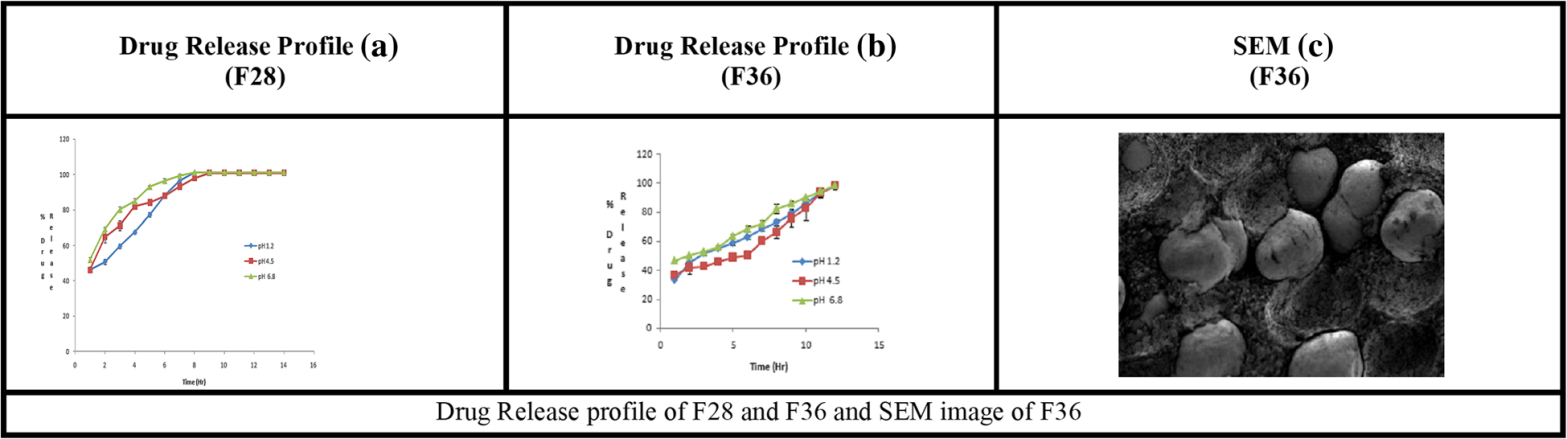
Drug Release profile of F28 and F36 and SEM image of F36

The combination pellets (F28) with the maximum concentrations of EC10FP, CW and GMS were also compacted into tablets (F36) at the optimized compression force of 5 kg, in order to control the burst release at 1st hour and drug release up to 12 h. After compaction, atenolol release at 1st hour (59.50% at pH 1.2, 71.17% at pH 4.5 and 80.07% at pH 6.8), was reduced to 33.5% (pH 1.2), 36.90% (pH 4.5) and 47.09% (pH 6.8). The compaction also prolonged the atenolol release from pellets, i.e. from 6 to 12 h (Fig. 6a and b). The pharmaceutical quality characteristics of compacted pellets (F36) are given in Table 4. The weight variation, hardness, friability disintegration time and content assay were found to be within the pharmacopeial limits.

**Table 4:** Physical properties of compacted matrix pellet formulation (F36)

	Weight variation (mg) (*n* = 20)	Disintegration Time (min) (*n* = 6)	Crushing Strength (Kg) (n = 20)	Assay (%) (*n* = 10)	Friability (%)
F36	130 ± 1.03	6.5 ± 0.50	5 ± 0.526	100.81	0.4

### Scanning Electron microscopy (SEM) of pellets

The cross-sectional scanning electron micrographs of F34, F35 showed cross-linking at 7000X magnification that was observed to be more dense and complex upon thermal treatment (Fig. 4c ii and 5c ii). The surface morphology of these polymers and wax based pellets was also smooth and spherical as shown in Fig. 4ci and 5ci. The smoothness, however, was found little more improved after thermal treatment Fig. 5ci, whereas, the overall appearance of both the formulations was similar as given in stereo images Fig. 4b and 5b. The SEM images and stereographs indicated that the surface morphology of the pellets was independent of the thermal treatment.

The SEM image Fig. 6c of compacted pellets (F36) showed that the integrity of pellets remained intact and become closer after the application of compression force.

#### Fourier transform infrared spectroscopy (FTIR):

**Fig. 7:**
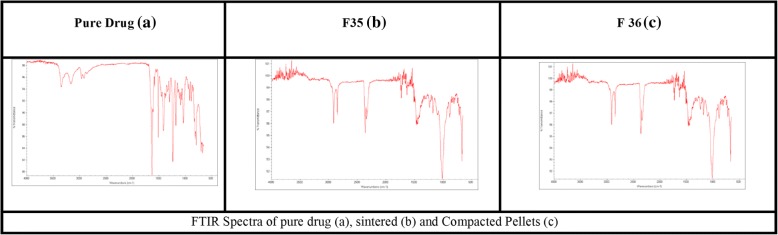
FTIR Spectra of pure drug (**a**), sintered (**b**) and (**c**) Compacted Pellets

Compatibility study among drug, polymers, and waxes, was performed using FTIR technique and the spectra exhibited absence of any interaction. The spectra of pure Atenolol at 3368 **cm**^**− 1**^ (-OH), 3198-3071 **cm**^**− 1**^ (H-N), 2966 **cm**^**− 1**^ (C-CH3), 2924 **cm**^**− 1**^ (CH2), 2870 **cm**^**− 1**^ (C-H), 1666 **cm**^**− 1**^ (C=O), 1649 **cm**^**− 1**^ (O=C-NH2),1614 **cm**^**− 1**^ Conjugated C=C (aromatic), 886 **cm**^**− 1**^ (C=CH2) are shown in (Fig. 7). In current study Fig. 7 (a, b and c) exhibits the FTIR spectra of pure atenolol, formulations F35 (sintered) and F36 (compressed matrix pellets), revealing no interaction between drug and excipients even after thermal treatment and compression force.

### Drug release kinetics

All trial formulations (F00-F36) were subjected to different release kinetic models (zero order, first order, Higuchi, Korsmeyer-Peppas, Hixson-Crowell, and Baker- Lonsdale) using DD solver (MS excel based Add-in program). Results revealed (Table 5) that Korsmeyer-Peppas was the best-fitted model to F35 at dissolution media pH 1.2, 4.5 and 6.8, i.e. r^2^ = 0.9975, r^2^ = 0.975 and r^2^ = 0.995 respectively, showing an-Fickian diffusion from polymeric lipid and cellulose based matrix system. F36 followed zero order kinetic release at pH 1.2 (r^2^ = 0.986) and 6.8 (r2 = 0.989), showing concentration independent release of Atenolol. Zero-order rate constant (k_0_) increased upon the application of compression force in all dissolution media. But at pH 4.5 the best-fitted model was Korsmeyer-Peppas (r^2^ = 0.971) showing, non-Fickian drug release.

**Table 5:**
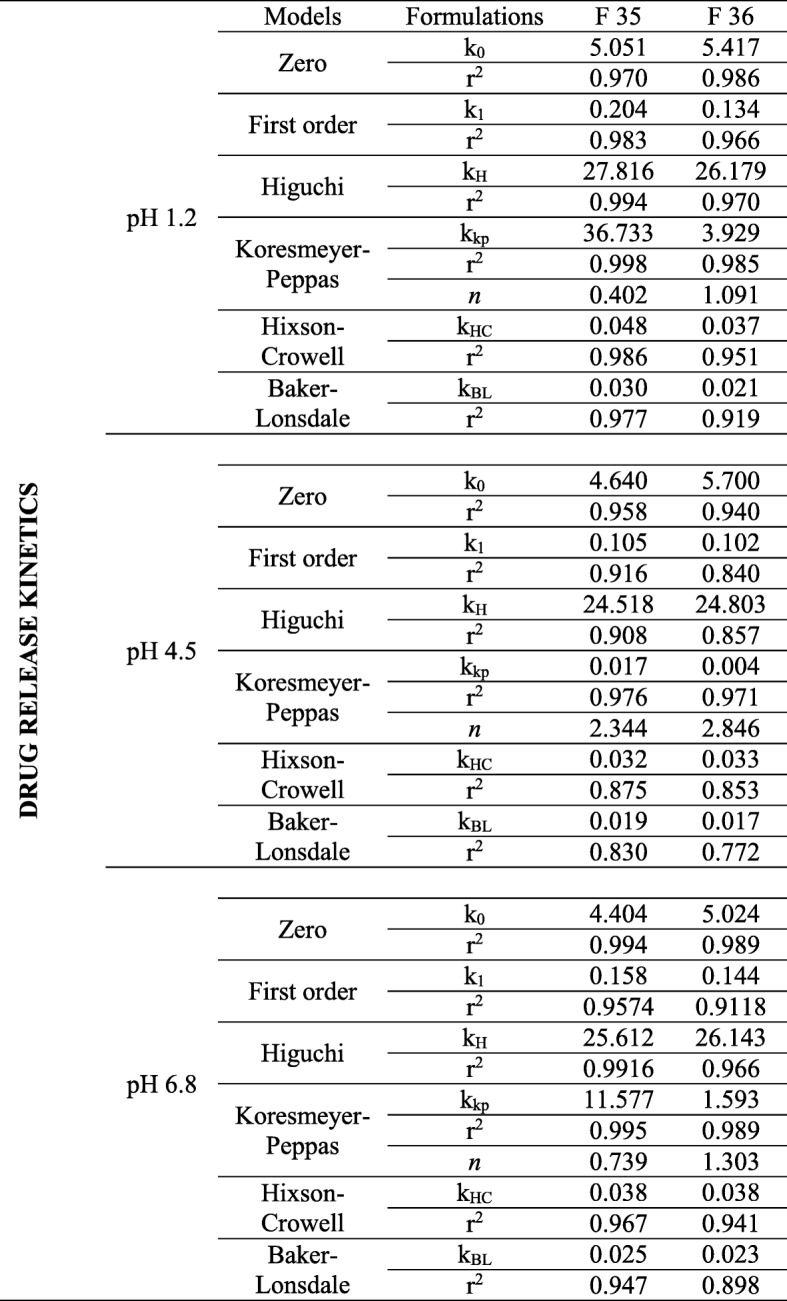
Drug release kinetics of optimized formulations

### Stability

The optimized Atenolol pellets (F35-F36) were stored according to ICH guidelines at 40^°^C/75% RH for 12 months. Percent assay and dissolution profiles at 3, 6, 9 and 12 months (Table 6) were determined. No significant difference was observed in surface morphology and release pattern of the drug. Statistical software Minitab (V: 17.0.1) was used to calculate the shelf life and found to be 43 months (F35) and 61 months (F36).

**Table 6:** Shelf life of optimized formulations

S. No	Formulations	Shelf life (months)
1	F35	43
2	F36	61

## Discussion

The extended release pellets of highly water soluble drug (BCS class III) Atenolol were designed by using response surface methodology (RSM) through software Design-Expert 10.0.8 (State-Ease, Inc., USA.) and prepared by extrusion and spheronization method. The effect of cellulose based polymers (HPMC K4, 15 & 100 M and EC 7, 10 & 100cps) and lipids (GMS &CW) alone and in combination, sintering and compaction, was observed on Atenolol release. Singh et al. in 2012 also used central composite design (CCD) and optimized SR pellets of Furosemide, to increase the bioavailability of the drug. For that purpose 1:3 ratio of drug and polymer (Coat L-100) along with the microcrystalline cellulose (MCC) were used and shaped by extrusion and spheronization. CCD used to optimize the drug while the process parameters were characterized by RSM. Results of dissolution carried out in USP apparatus I, indicated the significant difference between the drug release from SR pellet, commercial products and active [51].

Similarly, Thommes and Kleinebudde also worked on the physical characterization of pellets, containing kappa-carrageenan as an alternate to microcrystalline cellulose as pelletization aid, and reported the quality of pellets as independent of the type of filler and drug incorporated [52].

The different grades of HPMC (K4M, K15 M & K100 M) and concentration (5–205) with Atenolol produced dumbbell shaped pellets with less percentage yield and complete release of drug in the first hour. Nasiri et al. reported that all grades of HPMC failed to control the release of itopride hydrochloride (BCS class I drug) up to 12 h [35], similarly, Palmer et al. also observed the pH-independent release of ibuprofen from Poly Ethylene Oxide-HPMC matrix pellets [53].

Highest grade (EC 100FP) and concentration (20%), of ethyl cellulose (EC), failed to spheronize and retard the release of Atenolol from pellets with poor percentage yield. Dabbagh et al. reported that the rate of drug release decreased with the use of higher viscosity grades of EC, and reduction in particle size of polymer prolonged the release of Propranolol hydrochloride from ethyl cellulose-based matrix pellets, due to quick surface gel formation [54].

Due to high HLB value (3.8) [55], hydration and pore forming capability [56], GMS remained unable to control the release of Atenolol but showed best spherical pellets. Cheboyina and Wyandt stated that GMS pellets reduced drug release depending on physicochemical properties of the drug (such as solubility) [57].

Carnauba wax (CE) despite low wettability and hydrophobicity also failed to control the Atenolol release when used alone. The sphericity of pellets also became poor and irregular with CW [58]. Faaiza et al. reported that the CW is the best release retarding agent for poorly soluble drug- Meclizine HCl due to the absence of pores but destruct the morphology of pellets [59]. While, previously reported studies showed that the release of theophylline was retarded up to 3 h only and showed burst release was found even when CW was used in higher concentrations [4, 15].

Combination of ethyl cellulose (EC100FP) with waxes (GMS & CW), in the concentrations of 20, 20% & 10% respectively retarded drug release up to 12 h. This combination, however, remained unable to control the initial drug release at first hour, which was then controlled by thermal treatment. Upon heating CW releases from pellets and forms a hydrophobic waxy layer on to the pellets surface, this behavior participates in controlling drug release [15].

Compaction of Atenolol pellets containing EC 10FP (20%), GMS (20% and CW (10%), retarded drug release up to 12 h and controlled also controlled the initial burst release by reducing the surface area. Santos et al. also evaluated the compacted pellets of diclofenac sodium and ibuprofen composed of xanthan gum and characterized the drug release from tablets made from pellets [60].

The release of Atenolol showed different patterns with different formulation variables. All grades of cellulose polymers (HPMC & EC) (5–20%) released the drug completely within 4–5 h and unable to control the drug up to 12 h in the formulations. The same pattern was shown by GMS (10–50%) in the formulations. CW (5–20%) retarded the release up to 4 h only with the low initial release in comparison to GMS [4, 61].

The release of Atenolol was best suggested by Korsmeyer-Peppas model showing non-Fickian diffusion. The drug was dissolved through multiple mechanisms, leading to the softening of the matrix and followed by pore and channel formation. The same results were also reported by different researchers for different highly soluble drugs [55, 62, 63].

The SEM elaborated that sphericity and smoothness of EC100FP and lipids (GMS & CW) were superior. The Highest grade of EC and lipids (GMS & CW) form complex structure. Surface roughness and rigidity of pellets were controlled by the addition of GMS. Kleinebudde in 1997 introduced crystallite gel model of MCC in wet granulation, extrusion, and spheronization and reported that in the presence of water, MCC form a framework of cross-linking with hydrogen bonds, that results in the delicate network [64].

The IR frequency band of pure Atenolol has been reported by Eri et al. in 2014 [65]. The compatibility study showed no significant difference in the spectrum of pure Atenolol and that in optimized formulations. Only some additional peaks were observed before the spectra of Atenolol, might be associated with the different composition of CW. A researcher has also reported extra peaks of aluminum, copper, and zinc, in CW spectrum, due to its vegetable origin [59].

The stability of the optimized formulations was studied on accelerated conditions using ICH guidelines (40°C/75%RH) and found to be stable by means of the content assay and release profile. Kibria et al. reported anomalous drug release profile of ambroxol hydrochloride (highly soluble drug) when subjected to stability condition of 40° C/75%RH [66].

## Conclusion

The combination of CW (10%) GMS (20%) and EC 10FP (20%) in pellet formulation, could control the drug release of highly soluble drug after compaction for 12 h. On the other hand, the combination of EC100FP (20%), GMS (20%) and CW (10%) could control the burst release of drug at 1st hour after the thermal treatment of pellets at 90°C for 120 s. Atenolol is given both once daily and twice daily IR dosage forms in a different disease condition. Better designed once-daily extended release encapsulated or compacted Atenolol pellets is a better choice where patients will get controlled drug levels in the blood with a reduced side effect.

## Abbreviations

ANOVA: Analysis of variance
AR: Aspect Ratio
BCS: Biopharmaceutics Classification System
CCD: Central Composite Design
CW: Carnauba wax
EC: Ethyl cellulose
ER: Extended release
FTIR: Fourier Transform Infrared spectroscopy
GMS: Glyceryl monostearate
HPMC: Hydroxypropyl methylcellulose
ICH: International Conference on Harmonisation
MCC: Microcrystalline cellulose
PRESS: Predicted Residual Error Sum of Squares
RSM: Response surface methodology
SD: Standard Deviation
SEM: Scanning electron microscope
